# GeneTrail: A Framework for the Analysis of High-Throughput Profiles

**DOI:** 10.3389/fmolb.2021.716544

**Published:** 2021-09-16

**Authors:** Nico Gerstner, Tim Kehl, Kerstin Lenhof, Lea Eckhart, Lara Schneider, Daniel Stöckel, Christina Backes, Eckart Meese, Andreas Keller, Hans-Peter Lenhof

**Affiliations:** ^1^ Center for Bioinformatics, Saarland Informatics Campus, Saarbrücken, Germany; ^2^ Healthcare Digital & Data, Merck Healthcare KGaA, Darmstadt, Germany; ^3^ Department of Human Genetics, Saarland University, Homburg, Germany; ^4^ Chair for Clinical Bioinformatics, Saarland University, Saarbrücken, Germany; ^5^ Department of Neurology and Neurological Sciences, Stanford University School of Medicine, Stanford, CA, United States

**Keywords:** COVID-19, enrichment analysis, gene regulation, web server, time-serie analysis, single-cell analysis, network analyis, gene set analysis

## Abstract

Experimental high-throughput techniques, like next-generation sequencing or microarrays, are nowadays routinely applied to create detailed molecular profiles of cells. In general, these platforms generate high-dimensional and noisy data sets. For their analysis, powerful bioinformatics tools are required to gain novel insights into the biological processes under investigation. Here, we present an overview of the GeneTrail tool suite that offers rich functionality for the analysis and visualization of (epi-)genomic, transcriptomic, miRNomic, and proteomic profiles. Our framework enables the analysis of standard bulk, time-series, and single-cell measurements and includes various state-of-the-art methods to identify potentially deregulated biological processes and to detect driving factors within those deregulated processes. We highlight the capabilities of our web service with an analysis of a single-cell COVID-19 data set that demonstrates its potential for uncovering complex molecular mechanisms.

GeneTrail can be accessed freely and without login requirements at http://genetrail.bioinf.uni-sb.de.

## 1 Introduction

Modern high-throughput techniques enable detailed molecular profiling of hundreds of thousands of single cells. The resulting data sets are usually high-dimensional and noisy, making a manual inspection impossible. To facilitate the analysis of bulk- and single-cell data, various computational approaches have been developed that help to study the molecular signatures of the analyzed cells.

A common task in the analysis of molecular high-throughput profiles is the identification of biological processes that show differences between two groups of samples, e.g., disease versus control. For this purpose, three different generations are described in (Khatri et al., 2012): over-representation analysis (ORA), functional class scoring (FCS) procedures, and network-based methods. The first two classes, often referred to as enrichment analysis methods, use set-based statistics to check if biological categories are potentially deregulated without considering interactions between the considered molecular entities, e.g., genes. For the analysis of biological networks with a given pathway topology, network-based approaches have been developed that utilize the topology of these graphs to identify deregulated networks, signaling cascades, or subgraphs. Over the years, many tools have been developed that provide solutions for at least one of the three classes discussed above. The web services DAVID (Jiao et al., 2012), Enrichr (Kuleshov et al., 2016), and g:Profiler (Raudvere et al., 2019), for example, offer ORA-based approaches, whereas Babelomics (Alonso et al., 2015) is able to conduct different FCS and network-based approaches. WebGestalt (Liao et al., 2019) provides access to ORA, FCS procedures and network-based methods for a wide range of organisms. PaintOmics3 (Hernández-de Diego et al., 2018) offers solutions for ORA and network-based analyses and is capable of performing integrative analyses of multi-omics data sets. For a detailed review of existing tools and approaches see (Khatri et al., 2012; Das et al., 2020; Maleki et al., 2020).

In 2007, we launched the GeneTrail (Backes et al., 2007) web service that provided only enrichment analysis methods. Since then, it has been used in hundreds of thousands of analyses by many groups worldwide. Over the years, we have continuously extended its functionality and broadened the scope of application beyond traditional enrichment analysis, thereby creating an extensive framework for the integrative analysis of (epi-)genomics, transcriptomics, miRNomics, and proteomics data sets (Stöckel et al., 2016; Gerstner et al., 2020).

For our web service, we integrated 40 different external databases including biological categories from databases, like GO (The Gene Ontology Consort, 2021), KEGG (Kanehisa et al., 2021), and Reactome (Jassal et al., 2020). This comprehensive collection enables the analysis of putatively deregulated biological processes for 15 organisms. For their analysis, the toolbox currently offers well-established methods from a variety of enrichment and network analysis procedures. These include standard gene set based enrichment methods like over-representation analysis (ORA) (Drǎghici et al., 2003) and gene set enrichment analysis (GSEA) (Subramanian et al., 2007). For biological networks with a known topology, we also provide several methods to identify potentially deregulated networks, signaling cascades, or even subnetworks (Stöckel et al., 2013). Some of these approaches can also be used to identify molecular driving factors within those networks that may have induced the detected deregulation. In this context, we also offer a class of algorithms for the detection of transcriptional regulators that play essential roles in the investigated processes (Kehl et al., 2017).

Furthermore, in version 3.0, we added three specialized workflows that set GeneTrail apart from all other approaches (Gerstner et al., 2020). For the integrated analysis of various epigenetic modifications, we implemented a pipeline that detects biological processes affected by changes in the chromatin structure. Furthermore, our framework offers methods for exploring time-resolved expression signatures and identifying pathways whose activities change over time. Over the past years, advances in single-cell high-throughput methods shifted the focus of gene expression experiments from standard bulk samples to the investigation of thousands of individual cells. For this reason, we extended GeneTrail with a powerful single-cell analysis toolbox that offers functionality for the comparison of single cells of groups of cells (Gerstner et al., 2020).

## 2 Materials and Methods

GeneTrail is a comprehensive framework for the analysis of molecular high-throughput profiles with the goal to identify potentially deregulated biological processes and the driving molecular factors that might be responsible for these alterations. Our web service integrates the functionality of a variety of tools into one platform and thereby enables users to conduct different analyses on the same data set with minimal effort. Here, most of the core functionality is implemented as highly optimized C++ code that is available on GitHub (https://github.com/unisb-bioinf/genetrail3).

In all tools, users are guided through the analyses in an intuitive step-by-step manner. Here, automatic scripts analyze the properties of the input data and preselect suitable methods and parameters. This reduces the interactions required by the user and facilitates the analysis for non-experts. Furthermore, the different processing steps are well documented such that users can retrace the parameter choices and adapt them if needed. The analyses are supported by interactive visualizations ranging from broad overviews of the results to specialized in-depth representations.

**FIGURE 1:**
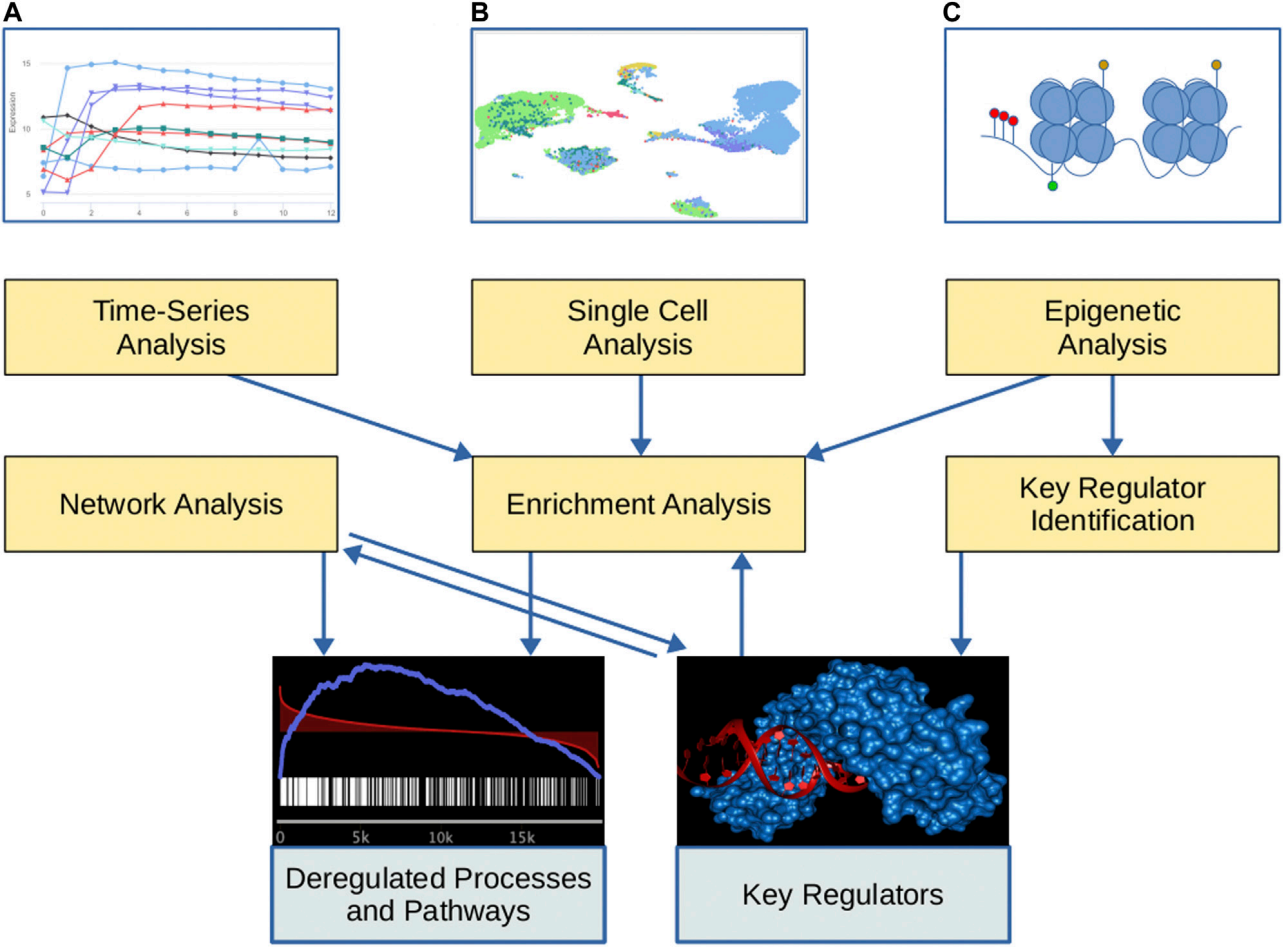
Overview of the GeneTrail tool suite. Yellow boxes represent the functionality of the tool suite and gray boxes represent the respective output. At the top, graphical representations of different input types are depicted. **(A)** Time-series expression data. **(B)** Single-cell expression data (UMAP representations of the cells). **(C)** Epigenetic data: Histone modifications (shown as green and yellow marks), open chromatin regions, and cytosine methylation (shown as red marks).

In order to fulfill the different tasks, we integrated biological knowledge from 40 different external databases. An overview of this data collection is depicted in Supplementary Material S1 and a graphical overview of the tool suite is given in Figure 1. In the following, we will provide a brief overview of the functionality of our web service. Although most methods can be applied to analyze measurements of genes, proteins, and miRNAs, we restrict the subsequent description to the gene-level to improve readability.

In many application scenarios, two groups of samples, e.g., disease versus control, are compared. For this purpose, a user can upload a gene expression matrix with samples from both groups, which can be analyzed for differential expression by our framework. To this end, our toolbox offers 15 different methods, including non-parametric measures, such as fold-changes or the Wilcoxon rank-sum test, but also parametric tests, like t-tests, DESeq2 (Love et al., 2014), edgeR (Robinson et al., 2010), or RUVSeq (Risso et al., 2014). Each of these methods produces a score per gene that mirrors the difference in expression. Additionally, GeneTrail provides several functions to optionally process the resulting scores. For example, scores can be transformed to absolute values if users are not interested in the direction of the expression changes. Additionally, the subsequent enrichment and network analysis methods require as input either a complete list of genes with assigned scores or a set of deregulated genes. In order to select these deregulated genes, our framework offers several filter procedures.

For DESeq2, edgeR, and RUVSeq, we used the respective R libraries. We implemented all remaining methods in C++ and the code is available in the GeneTrail C++ library (Stöckel et al., 2016) (see Supplementary Material S1).

### 2.1 Enrichment Analysis

An important task in the analysis of molecular high-throughput profiles is the identification of putatively deregulated biological processes, e.g., pathways that differ in activity between two sample groups.

The input for an enrichment analysis is either a small set of genes, e.g., the most differentially expressed ones, or a complete list of genes with scores that indicate their degree of deregulation. These data sets can either be directly uploaded to the web server or calculated based on a given gene expression matrix, as described above.

In addition to this input, enrichment methods require a set of biological processes or categories that should be analyzed. Here, GeneTail offers a large collection of categories that are extracted from external databases, covering nearly 65,000 gene-based categories for humans alone. These include biological processes, molecular functions, or cellular components from the Gene Ontology (The Gene Ontology Consort, 2021) and signaling pathways from KEGG (Kanehisa et al., 2021), Reactome (Jassal et al., 2020), or WikiPathways (Martens et al., 2021) (cf. Supplementary Material S1).

**FIGURE 2:**
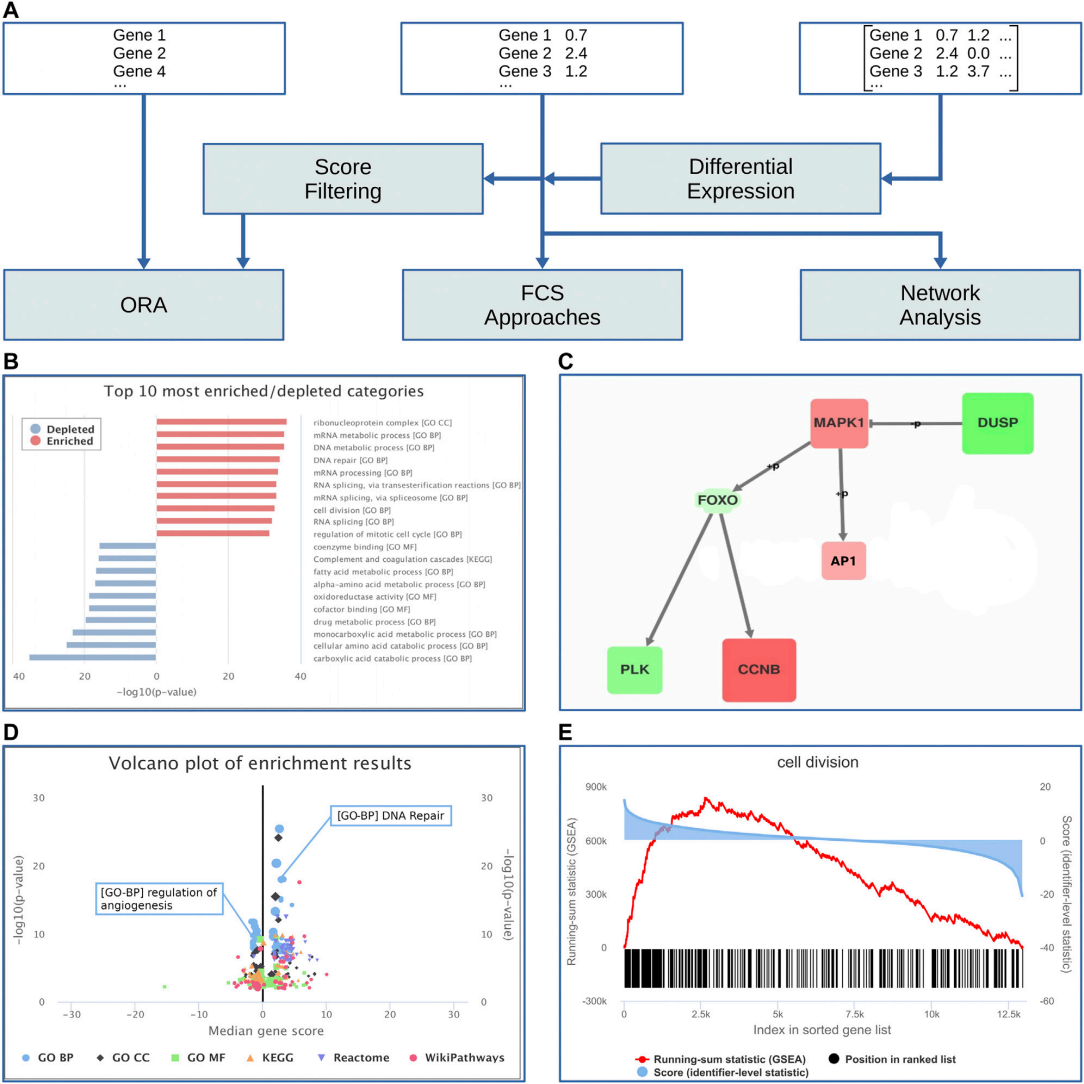
Schematic workflow of the enrichment and network analysis. **(A)** In this simplified flowchart, white boxes represent input types and gray boxes represent processing steps. Below the flowchart, examples of available visualizations are shown: **(B)** Overview representation of the top ten enriched and depleted categories. **(C)** Deregulated subnetwork with upregulated (red) and downregulated (green) nodes. **(D)** Volcano plot with two highlighted categories. **(E)** The GSEA result plot for an example category (“cell devision”) shows (i) the running-sum statistic in red, (ii) positions of member genes in the sorted gene list in black, and (iii) the corresponding gene scores in blue.

For the analysis of these categories, GeneTrail offers eleven enrichment algorithms that can be categorized into two classes. For a set of preselected genes, e.g., the most differentially expressed genes, an ORA can be applied to detect putatively deregulated biological processes. For complete gene lists with scores, such as fold-changes or t-scores, our web server also offers various FCS methods. These include non-parametric approaches that operate on the order of genes, like GSEA (Subramanian et al., 2007) or Wilcoxon rank-sum test. Additionally, we offer parametric tests that compare the scores of category members against genes that are not members of the category, such as the two-sample t-test or averaging methods that calculate summary statistics of category members (Ackermann and Strimmer, 2009). An overview of the different processing steps and available output visualizations is shown in Figure 2. We have implemented all enrichment methods in C++ (GeneTrail C++ library).

### 2.2 Analysis of Biological Networks

In contrast to enrichment-based methods that do not consider interactions between genes, network-based approaches utilize the topology of the corresponding interaction networks, e.g., provided by the KEGG database (Kanehisa et al., 2021), to identify potentially deregulated subnetworks or signaling cascades.

In addition to the given topology, network-based algorithms usually require a list of scores that for example mirror gene expression differences. These methods often use the given scores as weights for the vertices or the edges of the graph and usually try to identify subgraphs, paths, or signaling cascades that might be deregulated.

For this purpose, our toolbox provides two methods for the identification of the most highly altered subgraphs in biological networks: FiDePa (Keller et al., 2009) and an ILP approach (Backes et al., 2012). FiDePa uses a GSEA-like statistic to determine all paths in a given network enriched with differentially expressed genes. In addition to linear paths, the ILP approach is able to detect branched subgraphs with the highest degree of deregulation. Both approaches allow users to identify not only highly altered parts of networks, but also key molecules that might induce the detected deregulations within those subgraphs, e.g., the roots of the respective subnetworks. We have implemented both algorithms in C++.

### 2.3 Identification of the Most Influential Transcriptional Regulators

Driving elements in many biological processes are transcriptional regulators, like transcription factors, chromatin modifiers, or cofactors. Changes in the activity of these proteins can severely alter the expression of their target genes and, hence, deregulations of transcription factors are associated with certain diseases, e.g., cancer (Lee and Young, 2013). Therefore, an important goal in the analysis of deregulated biological processes is the identification of regulators that play key roles in these processes.

GeneTrail offers two classes of methods for the identification and prioritization of influential regulators (Kehl et al., 2017). The first class represents approaches, like REGGAE (Kehl et al., 2018), RIF1, and RIF2 (Reverter et al., 2010), that are based on experimentally validated regulator-target interactions (RTIs). In addition to RTIs, these algorithms require a gene expression matrix to find influential regulators that might contribute to gene expression differences between two sample groups.

Instead of experimentally validated binding sites, the second class uses predicted binding motifs of transcription factors in form of position weight matrices (PWMs) to study the binding patterns of these regulators. One of these methods is TEPIC, which combines open-chromatin regions, PWMs, and gene expression values in an integrative analysis to identify key regulators (Schmidt et al., 2019).

With the exception of the TEPIC framework that has been developed by Schmidt et al. (Schmidt et al., 2019), we implemented all other approaches in C++ as part of the GeneTrail C++ library.

### 2.4 Analysis of Time-Series Data

The structure and function of cells are controlled by complex networks of dynamic molecular mechanisms. Time-resolved expression profiles render it possible to study the dynamics of biological processes.

For the analysis of such time-resolved gene expression data, our framework offers methods to detect gene clusters with similar expression time courses and to identify molecular functions influenced by these clusters (Gerstner et al., 2020). To this end, GeneTrail conducts the following processing steps: First, the loaded expression data is filtered to select the genes with the highest expression change in the analyzed time frame. To this end, our framework provides different measures that assess the amount of change within the analyzed time frame, e.g., the aggregated expression difference between all consecutive time-points. The user can then define a threshold that is used to identify the genes with the strongest expression changes. The remaining genes are clustered with respect to their expression profiles. To this end, our framework provides a variety of similarity measures and clustering algorithms that are specifically tailored to the comparison of expression time curves. For the member genes of the resulting clusters, our web service conducts ORAs to detect molecular functions that are controlled by these clusters.

We implemented all processing steps either as R scripts or C++ programs that are part of the GeneTrail library.

### 2.5 Integrative Analysis of Epigenetic Modifications

The chromatin structure induced by epigenetic modifications, like cytosine methylations or histone marks, constitutes an essential regulatory layer of gene expression, and the investigation of chromatin states provides crucial information about the activity of cellular processes. To this end, GeneTrail offers functionality for the integrative analysis of histone modifications, open-chromatin regions, and DNA methylation patterns in different sample groups, e.g., disease versus control.

To start an analysis, users can upload epigenetic modifications for the samples in “bed,” “vcf,” or “idat” format. For each sample group, the tool suite first investigates the epigenetic modifications in the regulatory regions of all genes. Based on these modifications, each gene is assigned to one of four chromatin states: “active,” “poised,” “repressed,” or “no signal.” For this purpose, we use a rule-based approach, in which specific combinations of epigenetic marks define the chromatin state of a gene. The rules were manually curated from literature and the HIstome (Khare et al., 2012) and HHMD (Zhang et al., 2010) databases. A detailed description of this approach can be found in our online documentation.

Given the chromatin state of each gene, our toolbox clusters genes into transition groups. Here, a transition group is a group of genes that transition from a particular chromatin state in one sample group to another chromatin state in the second group, e.g., from a poised state in the disease group to an active state in the control group. For each transition group, ORAs are conducted to uncover changes of biological processes that are induced by this group.

For the initial processing of the uploaded files, we use BEDTools (Quinlan, 2014), biscuit (Zhou, 2016) and RnBeads (Müller et al., 2019). For all remaining steps, we use the functionality of GeneTrail’s C++ library.

### 2.6 Analysis of Single-Cell Data

Advances in high-throughput techniques enable the generation of molecular profiles for thousands or even hundreds of thousands of cells simultaneously. GeneTrail can analyze the resulting data sets to identify active biological processes for each cell and to detect functional changes between different cells, cell types, or groups of cells.

To start an analysis, the user has to provide a single-cell expression matrix and an associated metadata file that contains further information about each cell. This information may include sample identifiers, cell types, precomputed clusters, or assignments to certain classes of diseases. In a first step, GeneTrail removes cells that do not fulfill adjustable quality controls, e.g., damaged cells or duplets. The gene expression values of the remaining cells are then normalized and for each cell the genes with largest normalized expression are selected, e.g., the 500 most highly expressed genes. Based on the selected genes, active biological processes are identified for each individual cell via an enrichment analysis. Depending on the provided metadata, cells can be assigned to groups and differences in the activity of biological processes between the groups can be identified by GeneTrail. Additionally, our toolbox offers the Louvain algorithm (Que et al., 2015) for calculating clusters of the given cells. The identified clusters can also be used as group assignments.

Furthermore, our framework provides interactive UMAP (McInnes et al., 2018) and t-SNE (Van der Maaten and Hinton, 2008) visualizations of the calculated results.

For filtering, clustering and dimensionality reduction, we apply Seurat4 (Yuhan et al., 2021) and Monocle3 (Qiu et al., 2017). For all remaining analysis steps, we use the functionality of the GeneTrail C++ library.

## 3 Results and Discussion

The coronavirus disease 2019 (COVID-19) is a highly infectious respiratory illness caused by the severe acute respiratory syndrome coronavirus 2 (SARS-CoV-2) (Oran and Eric, 2020; Yuki et al., 2020). According to a study by Wu et al. (Wu and McGoogan, 2020), most affected individuals in the analyzed chinese cohort exhibit only mild-to-moderate symptoms, however, around 20% are categorized as severe or even critical cases. In these instances, the patients often show considerable alterations in the activity of the immune system, which includes highly elevated levels of pro-inflammatory cytokines. In current research, several factors are discussed that might cause these changes including pathogenic T-cells or inflammatory monocytes (Guo et al., 2020; Zhou et al., 2020).

Here, we demonstrate the capabilities of GeneTrail by investigating molecular processes that distinguish COVID-19 patients with acute respiratory distress syndrome (ARDS) from hospitalized patients that required no ventilation (NoVent), and healthy controls (Healthy). To this end, we analyze single-cell RNA-seq data of 10,339 CD14 monocytes that are part of a peripheral blood mononuclear cells (PBMCs) data set of Wilk et al. (Wilk et al., 2020). In total, the data set contains gene expression profiles of eight samples from seven hospitalized patients, of which four required mechanical ventilation, and six samples from healthy controls.

In particular, we applied GeneTrail to identify deregulated biological processes and corresponding key molecules that may contribute to the severity of COVID-19 cases with ARDS.

### 3.1 Identification of Deregulated Biological Processes in Single-Cell Expression Data

In the following, we discuss biological processes that show altered activities between the three investigated groups (ARDS, NonVent, Healthy). To this end, we use the single-cell functionality of our web service that first determines significantly enriched biological processes for each cell. We then tested for each category if it is significantly more active in one of the three groups (cf. Section 2.6). All processing steps and parameters are described in Supplementary Material S1 and the full results are shown in Supplementary Material S2.

**FIGURE 3:**
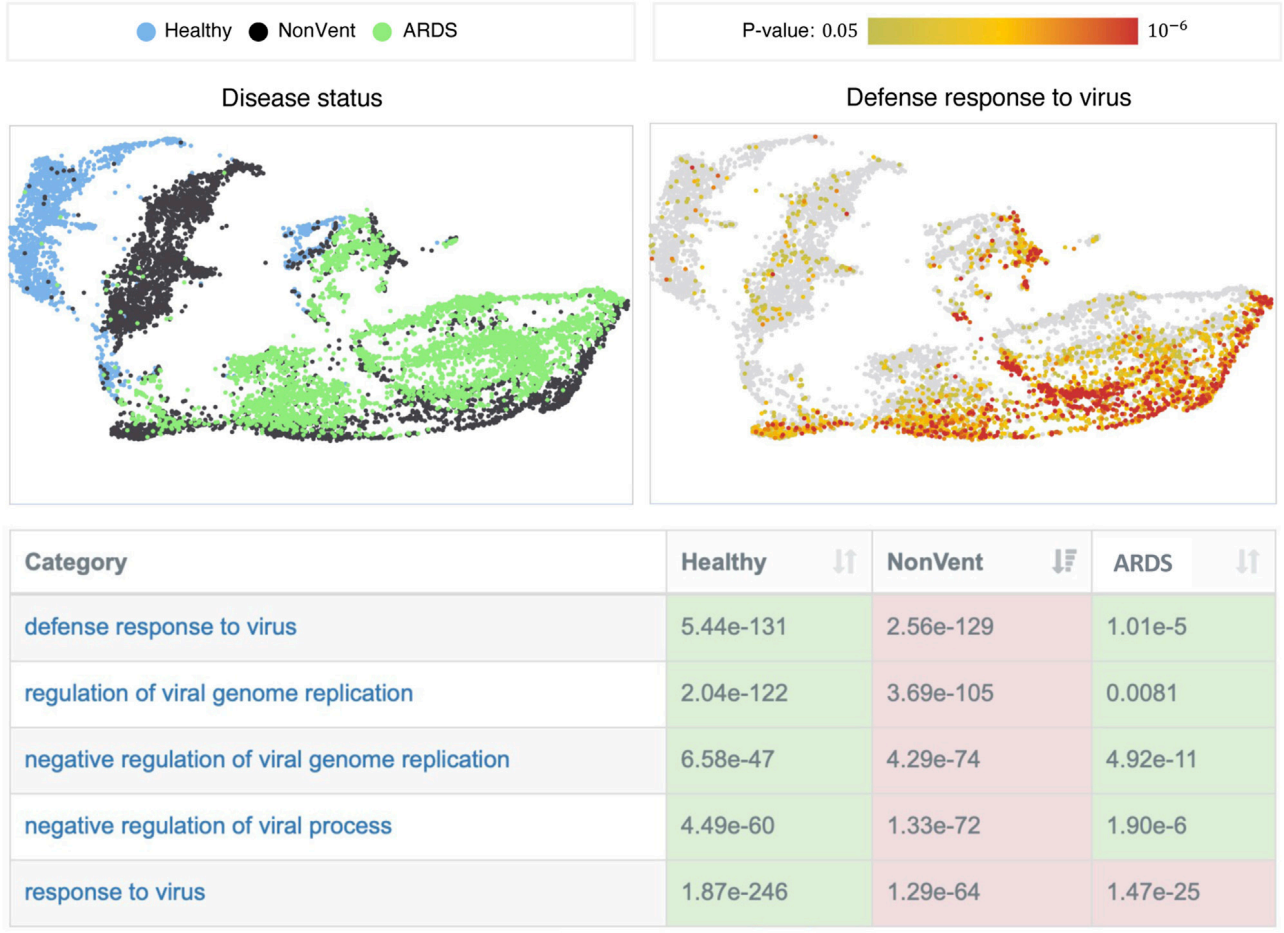
Overview of the single-cell result visualization. The table shows categories that are associated with “defense response to virus” and their *p*-values. The color indicates if a category is significantly more active (red) or inactive (green) in the corresponding group. The plots on the top show UMAP visualizations of the data set, where each point represents a single cell. The cells in the left image are colored according to their group membership and the cells in the right image are colored with respect to the *p*-values of the selected category (“defense response to virus.”)

We find the most significant differences in categories that are directly associated with the “defense response to virus” (cf. Figure 3). While these processes are inactive in healthy controls, they are highly active in the NonVent group, but show only a reduced activity in the ARDS group. The decreased activities of these processes in the ARDS group indicate that the adaptive immune response of ARDS patients could be impeded. A phenomenon that has been repeatedly reported in patients with severe courses of COVID-19 (Arunachalam et al., 2020; Janssen et al., 2021). We observe a similar behavior for biological categories involved in the response to type I, II, and III interferons and interferon signaling pathways, which are significantly more active in patients that required no ventilation. In particular, type I interferons (IFNs) and interferon-stimulated genes are crucial factors in antiviral processes. A deficiency of type I IFNs in patients with severe courses of COVID-19 has already been observed in several other studies (Acharya et al., 2020; Hadjadj et al., 2020; Lee and Shin, 2020) and is often accompanied by higher activities of tumor necrosis factor (TNF) production and NF-kappaB signaling. Our enrichment results confirm these observations. Furthermore, the decreased viral defense results in higher virus loads in the blood of ARDS patients (Hadjadj et al., 2020).

Additionally, we identified an increased endocytosis and phagocytosis activity in the ARDS group. These observations are also confirmed by enriched categories that are involved in macrophage activation. Increased activity of this process has even been described as a marker for the mortality in COVID-19 (Banu et al., 2020).

We also observe various enriched processes that indicate a highly increased motility, migration, and chemotaxis activity in the cells of the ARDS group. This might be linked to the increased TNF production in these cells, which is known to promote chemotaxis in monocytes (Ming et al., 1987).

A further factor that distinguishes the different groups is the activity of categories involved in antigen processing and presentation via MHC class II. The activity of these processes is significantly decreased in the ARDS group. This has also been described as a marker for the severity of COVID-19 (de Sousa et al., 2020; Liang et al., 2021). We also observe a significant decrease in expression of several MHC II components in the ARDS group, including HLA-DRA, which has been highly associated with severe respiratory failure in COVID-19 patients (Giamarellos-Bourboulis et al., 2020).

Moreover, many enriched categories indicate that the proliferation of cells in the ARDS group is significantly lower than in the NonVent group and in healthy controls.

In summary, our enrichment results suggest that central processes involved in the adaptive immune response to viruses might be impeded in the ARDS group, while the innate immune system seems to be overactive. These observations have also been made by several studies that compared mild and severe courses of disease (Arunachalam et al., 2020; Janssen et al., 2021).

We also analyzed the gene expression profiles of lymphocytes, i.e., B cells, T cells, and NK cells, and obtained similar results (cf. Supplementary Material S2).

### 3.2 REGGAE Analysis of Pseudo-bulk Expression Data

Based on the single-cell data set, we also created pseudo bulk expression data for each sample using the muscat R-package (Crowell et al., 2020). The resulting data was used to identify influential transcriptional regulators. For all genes, we first calculated expression differences between samples from the ARDS group and all other samples. In a second step, we selected the 250 most upregulated genes in the ARDS group and conducted a REGGAE analysis to find key regulators that have a strong influence on these 250 genes. The used parameters and the full results can be found in Supplementary Materials S1, S2 respectively.

**FIGURE 4:**
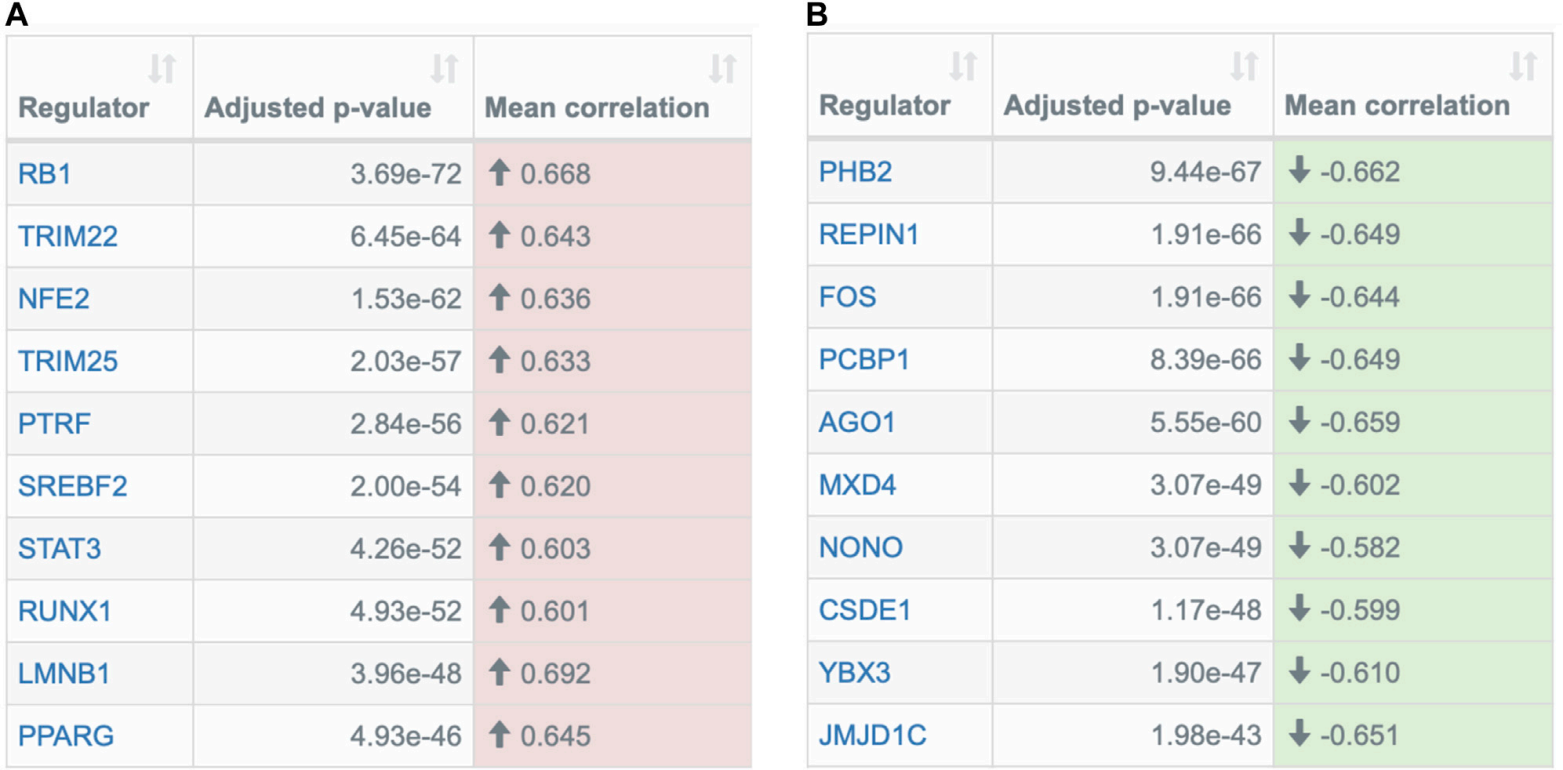
Overview of the most significant transcriptional regulators detected by the REGGAE algorithm. Depicted are the top ten potential **(A)** activators and **(B)** repressors. The first column in both tables contains the HGNC gene symbols of the regulators, the second column specifies the adjusted *p*-value, and the last column depicts the mean correlation of the regulator and all target genes in the analyzed gene list. The color indicates if the mean correlation is positive (potential activator) or negative (potential repressor).

The REGGAE analysis identified 171 regulators with significant influence, of which 56 are potential activators and 115 repressors. The ten most significant activators and repressors are shown in Figure 4. In general, many of the identified genes are key regulators of the immune system, e.g., members of the STAT family (Matsuyama et al., 2020) and the AP-1 complex (Zhu et al., 2021).

Additionally, among the top ten activators and repressors, some regulators are known to directly interact with proteins of SARS-CoV-1: RB1 (Bhardwaj et al., 2012), TRIM25 (Hu et al., 2017a), and PHB2 (Cornillez-Ty et al., 2009). Due to the sequence similarities of both corona viruses [79% (Suryawanshi et al., 2020)], these interactions may be conserved, but this requires further investigations.

Other regulators have already been discussed in the context of COVID-19. One of them is the transcription factor RUNX1 that plays a key role in many biological processes, in particular hematopoiesis (Okuda et al., 2001). RUNX1 has also been described as an important regulator in several diseases, including pulmonary diseases (Tang et al., 2018). It is investigated as a potential target molecule for therapy of pulmonary fibrosis (PF) in COVID-19 (O’Hare et al., 2021). In mouse models, it has been shown that the inhibition of RUNX1 successfully mitigates PF and reduces the expression of the host proteins ACE2 and FURIN, which mediate the SARS-CoV-2 infection (O’Hare et al., 2021).

Another regulator that has already been discussed as a potential marker for severe cases of COVID-19 and a putative therapy target is the transcription factor SREBF2. In general, SREBF2 regulates the lipid metabolism. This process is known to be vital for virus replications and members of the SREB family, including SREBF2, have been discussed as potential targets for aniviral strategies (Yuan et al., 2019). Lee et al. showed that SREBF2 is activated in PBMC samples of COVID-19 patients. Additionally, based on an infectious disease mouse model, Lee et al. demonstrated that an inhibition of SREBF2 suppressed cytokine storms and prevented pulmonary damages (Lee et al., 2020).

While many of the regulators have already been discussed in the context of SARS-CoV-1 and SARS-CoV-2, some of them might be interesting new candidates for further research.

### 3.3 Further Analyses

The results described in the last two sections clearly demonstrate that GeneTrail is well equipped for the identification of potentially deregulated biological processes and driving factors in humans.

However, our web service is not restricted to the analysis of human samples. In the past, it has been used in broad range of application scenarios from research groups around the world. Amongst others, it has been applied to study 1) differences in methylation patters in human and chimpanzee brains (Jia et al., 2012), 2) the molecular basis of heterosis in thale cress hybrids (Andorf et al., 2010), or 3) evolutionary differences between giant and red pandas (Hu et al., 2017b).

Moreover, in Supplementary Material S1, we provide an additional analysis of a thale cress (Arabidopsis thaliana) data set from Herranz et al. (2019). Here, we analyzed which biological processes in plant seedlings are affected by different light and gravity conditions on the International Space Station (ISS).

In order to present additional features of our web service, we also compiled several example analyses on our web sites, which contain step-by-step instructions, technical background information, and interpretation of the results.

## 4 Conclusion

Since the initial release of GeneTrail in 2007, we have continuously extended the functionality of our web service. In its current form, our tool suite provides various methods for the integrative analysis of multi-omics profiles. Our framework can be applied to study deregulated biological processes and their molecular driving factors in bulk, time-series, and single-cell data sets. For this purpose, it offers a variety of approaches for 1) enrichment analysis, 2) network analysis, and 3) the identification of key regulators.

Compared to other approaches, GeneTrails excels by providing rich functionality with highly efficient C++ implementations for a broad range of application scenarios. The provided approaches can be used to analyze a comprehensive collection of biological categories and pathways that stem from 40 different biological databases and 15 organisms. Additionally, the rich functionality of our web service is complemented with an intuitive web interface that offers many interactive visualizations ranging from a broad overview of the results to detailed in-depth representations.

We demonstrated GeneTrail’s capabilities by analyzing single-cell expression profiles of CD14 monocytes from COVID-19 patients and healthy controls. Our tools identified many processes that show different activities between the three considered groups (ARDS, NonVent, and Healthy). In particular, our results indicated that the activity of the adaptive immune response in the ARDS group might be reduced, while processes of the innate immune response seem to be overactive. Here, many of our observations have already been discussed in literature.

Moreover, we analyzed key transcriptional regulators that have a strong influence on the most upregulated genes in the ARDS group. Amongst them, we not only identified several regulators that are already known as markers for the severity of COVID-19 but also potential candidates that require further research.

In the future, we will continue to extend our framework with new analysis functionality for the identification of regulatory interactions and support for single-cell multimodal omics data (Teichmann and Efremova, 2019), which may provide a deeper understanding of the biological processes under investigation. Still, the current rich functionality of our web server combined with the intuitive web interface and interactive visualizations already make GeneTrail one of the most comprehensive tool suites for the analysis of molecular high-throughput profiles and set it apart from other approaches.

## Data Availability

Publicly available datasets were analyzed in this study. This data can be found here: https://www.ncbi.nlm.nih.gov/geo/query/acc.cgi?acc=GSE150728 GEO Accession: GSE150728 and the code to preprocess the dataset is available at https://github.com/unisb-bioinf/GeneTrail-tool-suite-COVID19-case-study.
