# Supplementary material for: GeneTrail: A Framework for the Analysis of High-Throughput Profiles
Source: Front Mol Biosci. 2021 Sep 16;8:716544. doi: 10.3389/fmolb.2021.716544 (PMC8481803; doi:10.3389/fmolb.2021.716544)
Supplement: Supplementary file 1 [file DataSheet1.PDF]

# - Supplement S1 -

1 COVID-19 single-cell data set

The COVID-19 single-cell data set analyzed in the paper was published by Wilk et al. (1). It contains expression data for 44,722 cells and 14 samples (four ARDS samples, four NonVent samples, and six healthy controls). The data was generated using the Seq-Well (3) sequencing platform for scRNA-Seq. The generated reads were aligned to the human reference genome GRCh37 using STAR (55), and then, count matrices were created with dropEst (5). Afterwards, cells of low quality were removed. This includes cells for which the sum of UMIs was fewer than 1,000 or more than $15,000$ or for which mitochondrial genes or rRNA genes made up for more than 20% of total UMIs. Additionally, potential cell duplets were removed. To this end, cells were excluded from further analysis, for which an unusually high number of genes were detected in comparison to the total amount of UMIs in the cell. In more detail, cells were removed for which more than 75 genes were detected per 100 UMIs. Finally, only genes expressed in more than nine cells are kept in the matrix. All of these preprocessing steps were conducted by Wilk et al.

1.1 Data processing

We downloaded the pre-filtered count matrix and associated metadata for each cell from the COVID-19 Cell Atlas hosted by the Wellcome Sanger Institute (dataset with name "Peripheral Blood Mononuclear Cells (PBMCs)" in the "Patient donors" tab). For our analysis, we only considered the gene expression profiles of CD14 monocytes, which resulted in a final data set of 10,339 cells. This final data set is available for download on the GeneTrail single-cell analysis start page.

2 Identification of deregulated biological processes in single-cell expression data

In addition to the preprocessing done by Wilk et al., we conducted the following processing steps for the raw count matrix of CD14 monocytes with our tool suite.

2.1 Identifier mapping

First the identifier for all genes in the data set were mapped to Official gene symbols.

Parameter	Value
Method to remove duplicates	median
Database Version	V3
Tool Version	V3.2

2.2 Quality control

We then applied several filter criteria to the matrix to remove cells with insufficient quality.

Parameter	Value
Minimum number of UMIs	500
Minimum number of expressed genes	500
duplicateMethod	median
Database Version	V3
Tool Version	V3.2

2.3 Normalization

For cells that pass the quality filters, we normalized the expression of all genes.

Parameter	Value
Normalization method	log2(RPM+1)
Database Version	V3
Tool Version	V3.2

2.4 Feature selection

From the normalized expression matrix, we then selected the most expressed genes for each cell.

Parameter	Value
Selection method	The X most highly expressed genes
Number of selected genes per cell	500
Database Version	V3
Tool Version	V3.2

2.5 Enrichment analyis

For each cell, we then conducted over-representation analyses using the selected genes.

Parameter	Value
Method for multiple testing correction	Benjamini-Yekutieli
Minimum number of category members	0
Maximum number of category members	700
Null hypothesis	upper-tailed
Significance level	0.05
Database Version	V3
Tool Version	V3.2

2.6 Dimension reduction

For the visualization of the results, we calculated UMAP coordinates with the Seurat package.

Parameter	Value
Most variable genes	2000
Database Version	V3
Tool Version	V3.2

3 Identification of key regulators

For the identification of key transcriptional regulators we conducted the following processing steps.

3.1 Pseudo-bulk computation

The pseudo-bulk expression data was generated based on the raw count matrix of CD14 Monocytes using the muscat R-package (Version 1.5.4) (2).

Parameter	Name
Method	`aggregateData`
x	the normalized Data
assay	NULL
by	c('cluster_id', 'sample_id')
fun	'sum'
scale	FALSE
verbose	TRUE
BPPARAM	SerialParam(progressbar = T))
Database Version	V3
Tool Version	V3.2

3.2 Normalization

The pseudo-bulk data set was then normalized.

Parameter	Value
Normalization method	Median library size per sample
Transformation	log2(x+1)
Database Version	V3
Tool Version	V3.2

3.3 Group comparison and feature selection

For all genes in the normalized pseudo-bulk, we then calculated expression differences between samples from the ARDS group and all other samples. In a second step, we selected the 250 most upregulated genes in the ARDS group.

Parameter	Value
Method for group comparison	log2 fold-change
sample group	C1B, C3, C4, C6
reference group	C1A, C2, C5, C7, H1, H2, H3, H4, H5, H6
Database Version	V3
Tool Version	V3.2

3.4 REGGAE analysis

For the top 250 most upregulated genes in the ARDS group, we then conducted a REGGAE analysis to identify key regulators that influence these genes.

Parameter	Value
Order in which test set is sorted	decreasingly
Order in which associations are sorted	absolute-decreasingly
Enrichment algorithm	Wilcoxon rank-sum test
Association score	Pearson correlation
Random seed	5662943078631823136
Number of bootstrapping runs	1000
Method to calculate confidence intervals	Percentile
Method for multiple testing correction	Benjamini-Yekutieli
Database Version	V3
Tool Version	V3.2

4 GeneTrail analysis of Arabidopsis thaliana exposed to different light and gravity conditions

In order to demonstrate the ability of GeneTrail to identify potentially deregulated biological processes in plants, we here describe an analysis of a recent *Arabidopsis thaliana* RNA-seq data set by Herranz et al. (53). In their study, *Arabidopsis thaliana* seedlings were exposed to blue light and different gravity levels in order to study the influence of both stimuli to the transcriptome. Our goal is to support their findings with enrichment analyses perfomed by GeneTrail.

4.1 Motivation

The growth of plants is affected by many environmental factors, including light, water, nutrients, and gravity. Especially for the orientation of plant roots, gravity and light are essential factors (52). Therefore, cultivating plants in environments with lowered gravity levels, e.g., mars or a space station, might have a negative effect on plant growth. Indeed, it was shown by several groups that plants grown in lower gravity levels have a reduced ribosome biogenesis and therefore a reduced biomass production (58; 59).
In a recent study by Herranz et al. (53), *Arabidopsis thaliana* seedlings were grown in the International Space Station (ISS) with varying gravity levels including micro gravity, moon gravity ($0.18g$), mars gravity ($0.36g$), reduced earth gravity ($0.57g$), and a regular earth gravity control ($1g$). Additionally, seedlings were stimulated with blue light, a novel technique that tries to counteract the negative influence of a lowered gravity level on plant growth. The aim of the study is to analyze the influence of lower gravity levels on the transcriptome and on biological processes in blue light stimulated *Arabidopsis thaliana* seedlings.

4.2 Analysis

4.2.1 Preprocessing

The data set by Herranz et al. contains expression data for 17 *Arabidopsis thaliana* seedling samples (four micro gravity samples, three moon gravity samples, three mars gravity samples, four reduced earth gravity samples, and three control samples). The data was generated using the Illumina HiSeq2500 sequencer. The resulting paired-end reads were trimmed using Trim Galore! (54) and then aligned to an *Arabidopsis thaliana* STAR reference (based on the reference genome TAIR10) using STAR (55). Afterwards, count matrices were created with RSEM (56) and normalized using DESeq2 (57). The resulting normalized expression data set is available at the GeneLab database with accession GLDS-251 (https://genelab-data.ndc.nasa.gov/genelab/accession/GLDS-251/). All of these preprocessing steps were conducted by Herranz et al.

4.2.2 Analysis with GeneTrail

In order to identify biological processes that are affected by the change in gravity level, we performed enrichment analyses with the GeneTrail web service. To this end, we downloaded the normalized *Arabidopsis thaliana* data set described above from the GeneLab database. Next, we performed a GeneTrail enrichment analysis for each gravity level except for the control as follows: We uploaded

the data set to the transcriptomics workflow and selected all samples of the respective gravity level as sample group. Additionally, all samples from the control gravity level were selected as reference group (for all four enrichment analyses). In order to calculate gene expression difference between the sample and the reference group, we selected the independent shrinkage t-test. As enrichment analysis method, GSEA was selected to find potentially enriched Gene Ontology (GO) and KEGG categories. The parameters used for the four enrichment analyses are listed in the tables below. Afterwards, we compared the four enrichment analysis results with the comparison functionality on the results page.

Parameters for differential gene expression analysis:

Parameter	Value
Should the input be annotated	true
Method to remove duplicate gene entries	median
Method for differential expression analysis	independent-shrinkage-t-test
Database Version	V3
Tool Version	V3.2

Parameter for the enrichment analysis:

Parameter	Value
P-value adjustment	benjamini_yekutieli
Adjust categories separately	true
Category databases	GO-BP, GO-CC, GO-MF, KEGG
Method to remove duplicate gene entries	median
Maximum category size	700
Minimum category size	2
Sort order	decreasing
Number of permutations	1000000
pValueStrategy	row-wise
seed	5200410782971513573
significance	0.05
Database Version	V3
Tool Version	V3.2

4.3 Results

The goal of our analyses is to confirm the results by Herranz et al. on ribosome biogenesis, chloroplasts and mitochondria. In this section, we discuss the different analyses and highlight supportive evidence for the findings of Herranz et al.

4.3.1 Ribosome biogenesis

Previous studies on *Arabidopsis thaliana* in low gravity levels or micro gravity levels reported a decreased ribosome biogenesis (58; 59). In this study, the seedlings were stimulated with blue light in order to reduce this negative effect. In their analysis, Herranz et al. found several potentially enriched

GO - Biological Process

Name	0.57g vs 1g	0.36g vs 1g	0.18g vs 1g	ug vs 1g
translation	8.00e-16	4.80e-24	9.36e-37	3.78e-37
peptide metabolic process	2.37e-13	8.15e-23	8.25e-37	3.78e-37
peptide biosynthetic process	5.01e-16	1.96e-24	8.25e-37	3.78e-37
cellular biosynthetic process	4.27e-4	8.47e-12	4.53e-18	1.29e-20
biosynthetic process	0.0014	4.43e-11	2.16e-15	8.68e-18

Previous 1 Next

Figure 1: Comparative enrichment result view of GeneTrail. Shown are the five categories from GO - Biological Process that are predicted as enriched in all four analyses. From left to right, the columns represent the q-values for the comparison of the control gravity level versus (1) reduced earth gravity, (2) mars gravity, (3) moon gravity, and (4) micro gravity.

biological pathways related to ribosome biogenesis, which might indicate a positive influence of blue light for plant growth.

As can be seen in Figure 1, our GeneTrail analysis resulted in five biological processes from GO Biological Processes (GO-BP) that were consistently predicted as enriched in the different gravity levels. These processes relate to an increased ribosome biogenesis (”translation”, ”peptide biosynthetic process”, ”cellular biosynthetic process”, and ”biosynthetic process”), and to an increased metabolic rate (”peptide metabolic process”). These findings are supported by two consistently upregulated pathways from KEGG, which also show an increased ribosomal activity (”Ribosome”) and an increased metabolic rate (”Oxidative phosphorylation”). The results for the KEGG pathways can be seen in Figure 2. These findings support the results from Herranz et al. and might suggest that stimulation with blue light counteracts the negative effect of low gravity levels on plant growth. A similar effect was previously achieved by stimulating *Arabidopsis thaliana* seedlings with red light in different gravity levels (60).

4.3.2 Chloroplast function

Chloroplasts are plastids in plant and algal cells that are able to conduct photosynthesis and are, hence, crucial for plant growth (64). In previous studies of plants grown in micro gravity, the func-

KEGG - Pathways

Name	0.57g vs 1g	0.36g vs 1g	0.18g vs 1g	ug vs 1g
Ribosome	↑ 1.35e-20	↑ 6.37e-38	↑ 6.37e-38	↑ 6.37e-38
Oxidative phosphorylation	↑ 5.76e-4	↑ 4.64e-22	↑ 2.49e-11	↑ 8.21e-16

Previous 1 Next

Figure 2: Comparative enrichment result view of GeneTrail. Shown are the two categories from KEGG that are predicted as enriched in all four analyses. From left to right, the columns represent the q-values for the comparison of the control gravity level versus (1) reduced earth gravity, (2) mars gravity, (3) moon gravity, and (4) micro gravity.

tions of chloroplasts seemed to be hampered in lower gravity levels (61; 62). This finding was also confirmed by Herranz et al. and the results of our GeneTrail analysis, in which biological processes for chloroplast function are consistently predicted to be depleted in lower gravity levels compared to earth gravity. In total, five potentially downregulated categories related to chloroplast function were found by GeneTrail for GO Cellular Compartment (GO-CC), as shown in Figure 3.

Three of the five potentially downregulated categories directly relate to thylakoids, which are compartments in chloroplasts responsible for all light-stimulated reactions. The three categories are "chloroplast thylakoid", "chloroplast thylakoid membrane", and "chloroplast thylakoid lumen". Interestingly, the significance of the results consistently declines with lowering gravity level, however rises again for micro gravity.

Additionally, two categories were predicted to be downregulated in lower gravity levels. These categories are related to the structure of chloroplasts ("chloroplast envelope") and to genes in the stroma of chloroplasts ("chloroplast stroma"). These results are expected as previous studies found the shape of chloroplasts to be significantly altered under lower gravity levels and also describe a potential malfunctioning of chloroplasts in micro gravity environments (61; 62; 63).

4.3.3 Mitochondria

Mitochondria are compartments with a double membrane and are capable of producing ATP. However, in contrast to chloroplasts, which are also able to produce ATP, categories related to mitochondria function were predicted as enriched in lower gravity levels by Herranz et al. Indeed, this result is confirmed by the comparative enrichment analysis of GeneTrail (cf. Figure 4). Although not as consistent as for categories related to ribosome biogenesis, categories related to mitochondria have a decreasing significance with decreasing gravity level. An exception is the mars gravity level, for which the categories related mitochondria were lowest compared to the other gravity levels.

In summary, the enrichment results obtained from GeneTrail point towards a potential of blue light to circumvent the negative influence of lowered gravity levels on the growth of *Arabidopsis thaliana*

GO - Cellular Component

Name	0.57g vs 1g	0.36g vs 1g	0.18g vs 1g	ug vs 1g
chloroplast thylakoid membrane	5.99e-13	1.00e-26	6.34e-34	5.54e-12
chloroplast envelope	1.42e-14	1.55e-25	2.31e-26	6.17e-10
chloroplast stroma	6.41e-17	7.69e-19	2.78e-23	8.09e-8
chloroplast thylakoid	4.31e-11	4.00e-14	2.01e-18	1.21e-7
chloroplast thylakoid lumen	1.01e-5	2.10e-7	2.51e-12	0.0265

Previous 1 Next

Figure 3: Comparative enrichment result view of GeneTrail. Shown are the five categories from GO - Cellular Compartment that are predicted as depleted in all four analyses. From left to right, the columns represent the q-values for the comparison of the control gravity level versus (1) reduced earth gravity, (2) mars gravity, (3) moon gravity, and (4) micro gravity.

GO - Cellular Component

Name	0.57g vs 1g	0.36g vs 1g	0.18g vs 1g	ug vs 1g
inner mitochondrial membrane protein complex	0.0047	2.88e-19	1.05e-12	5.34e-14
mitochondrial respiratory chain complex I	0.0054	3.69e-15	3.79e-9	3.18e-8

Previous 1 Next

Figure 4: Comparative enrichment result view of GeneTrail. Shown are the two categories from GO - Cellular Compartment related to mitochondrial activity. From left to right, the columns represent the q-values for the comparison of the control gravity level versus (1) reduced earth gravity, (2) mars gravity, (3) moon gravity, and (4) micro gravity.

seedlings. However, the interaction of gravity and light sensing on a molecular level still needs to be elucidated further in order to successfully cultivate *Arabidopsis thaliana* in lower gravity levels.

5 External libraries used in the GeneTrail C++ library

The GeneTrail C++ library is available at https://github.com/unisb-bioinf/genetrail3 and is based on several external libraries and programs depicted in the table below.

Library or program	Used by
boost C++ library (v.1.65.1) (51)	All programs
Eigen (v3) (48)	All parts of the library
cplex (v12.6.2) (50)	To solve the subgraph ILP in the network analysis
googletest (v1.8.0)	Most parts of the library
GNU parrallel (v2016-12-22) (49)	To compute ORAs in parallel for the single-cell pipeline

6 Overview of external databases

Content	Class	Integrated databases
Biological category	Pathways and Ontologies	Gene Ontology (GO)(6), KEGG(7), Reactome(8), Wikipathways(9), BioCarta(38), HumanCyc(10), NCI PID (40), PANTHER(12), MSigDB(13), ConsensusPathDB(14), NIA Phenotypes (41)
	Protein Complexes/Families	CORUM(15), EpiFactors(16), Pfam(17)
	SNPs	GWAS Catalog(18), PheWAS Catalog (42)
	Sequence features	NCBI Assembly (43)
	Cell type and tissue markers	The Human Protein Atlas(19), CellMarker(20)
	Disease signatures	PharmGKB(21), The Human Protein Atlas (19), MSigDB (13)
	miRNA	miRTarBase(22), miRPathDB(23)
Regulator binding information	Experimentally validated RTIs	TRANSFAC(24), ChIP-Atlas(25), ChIPBase(26), ENCODE(27), JASPAR(28), SignaLink(29), ChEA(30)
	PWMs	TRANSFAC (24), HOCOMOCO(31), JASPAR(28), ENCODE Motif Database (Kellis Lab) (45)
Identifer Mappings		Ensembl(32), NCBI Gene (46), UniProt(33), miRBase(34), miRTarBase (22), dbSNP (39)
Genomic Annotations		NCBI RefSeq(35), GENCODE(36), GeneHancer(37), T2T(47)

Table 1: Overview of the databases integrated into the GeneTrail tool suite. The second column depicts the type of information stored in the integrated databases that are shown in the third column. Some databases are listed for more than one information type, as they have multiple purposes (e.g., TRANSFAC).

References

[1] Wilk, Aaron J., et al. "A single-cell atlas of the peripheral immune response in patients with severe COVID-19." Nature medicine 26.7 (2020): 1070-1076.

[2] Crowell, Helena L., et al. "muscat detects subpopulation-specific state transitions from multi-sample multi-condition single-cell transcriptomics data." Nature communications 11.1 (2020): 1-12.

[3] Gierahn, Todd M., et al. "Seq-Well: portable, low-cost RNA sequencing of single cells at high throughput." Nature methods 14.4 (2017): 395-398.

[4] Dobin, Alexander, et al. "STAR: ultrafast universal RNA-seq aligner." Bioinformatics 29.1 (2013): 15-21.

[5] Petukhov, Viktor, et al. "dropEst: pipeline for accurate estimation of molecular counts in droplet-based single-cell RNA-seq experiments." Genome biology 19.1 (2018): 1-16.

[6] Gene Ontology Consortium. "The gene ontology resource: 20 years and still GOing strong." Nucleic acids research 47.D1 (2019): D330-D338.

[7] Kanehisa, Minoru, et al. "KEGG: integrating viruses and cellular organisms." Nucleic acids research 49.D1 (2021): D545-D551.

[8] Jassal, Bijay, et al. "The reactome pathway knowledgebase." Nucleic acids research 48.D1 (2020): D498-D503.

[9] Martens, Marvin, et al. "WikiPathways: connecting communities." Nucleic Acids Research 49.D1 (2021): D613-D621.

[10] Romero, Pedro, et al. "Computational prediction of human metabolic pathways from the complete human genome." Genome biology 6.1 (2005): 1-17.

[11] Schaefer, Carl F., et al. "PID: the pathway interaction database." Nucleic acids research 37.suppl_1 (2009): D674-D679.

[12] Mi, Huaiyu, et al. "PANTHER version 16: a revised family classification, tree-based classification tool, enhancer regions and extensive API." Nucleic acids research 49.D1 (2021): D394-D403.

[13] Liberzon, Arthur, et al. "The Molecular Signatures Database Hallmark Gene Set Collection." Cell systems 1.6 (2015): 417-425.

[14] Herwig, Ralf, et al. "Analyzing and interpreting genome data at the network level with ConsensusPathDB." Nature protocols 11.10 (2016): 1889-1907.

[15] Giurgiu, Madalina, et al. "CORUM: the comprehensive resource of mammalian protein complexes—2019." Nucleic acids research 47.D1 (2019): D559-D563.

[16] Medvedeva, Yulia A., et al. "EpiFactors: a comprehensive database of human epigenetic factors and complexes." Database 2015 (2015).

[17] Mistry, Jaina, et al. "Pfam: The protein families database in 2021." Nucleic Acids Research 49.D1 (2021): D412-D419.

[18] Buniello, Annalisa, et al. "The NHGRI-EBI GWAS Catalog of published genome-wide association studies, targeted arrays and summary statistics 2019." Nucleic acids research 47.D1 (2019): D1005-D1012.

[19] Uhlen, Mathias, et al. "A pathology atlas of the human cancer transcriptome." Science 357.6352 (2017).

[20] Zhang, Xinxin, et al. "CellMarker: a manually curated resource of cell markers in human and mouse." Nucleic acids research 47.D1 (2019): D721-D728.

[21] Whirl-Carrillo, Michelle, et al. "Pharmacogenomics Knowledge for Personalized Medicine." Clinical Pharmacology & Therapeutics 92.4 (2012): 414-417.

[22] Huang, Hsi-Yuan, et al. "miRTarBase 2020: updates to the experimentally validated microRNA–target interaction database." Nucleic acids research 48.D1 (2020): D148-D154.

[23] Kehl, Tim, et al. "miRPathDB 2.0: a novel release of the miRNA Pathway Dictionary Database." Nucleic acids research 48.D1 (2020): D142-D147.

[24] Matys, Volker, et al. "TRANSFAC® and its module TRANSCompel®: transcriptional gene regulation in eukaryotes." Nucleic acids research 34.suppl_1 (2006): D108-D110.

[25] Oki, Shinya, et al. "ChIP-Atlas: a data-mining suite powered by full integration of public ChIP-seq data." EMBO reports 19.12 (2018): e46255.

[26] Zhou, Ke-Ren, et al. "ChIPBase v2.0: decoding transcriptional regulatory networks of non-coding RNAs and protein-coding genes from ChIP-seq data." Nucleic acids research (2016): gkw965.

[27] Davis, Carrie A., et al. "The Encyclopedia of DNA elements (ENCODE): data portal update." Nucleic acids research 46.D1 (2018): D794-D801.

[28] Fornes, Oriol, et al. "JASPAR 2020: update of the open-access database of transcription factor binding profiles." Nucleic acids research 48.D1 (2020): D87-D92.

[29] Fazekas, Dávid, et al. "SignaLink 2–a signaling pathway resource with multi-layered regulatory networks." BMC systems biology 7.1 (2013): 1-15.

[30] Lachmann, Alexander, et al. "ChEA: transcription factor regulation inferred from integrating genome-wide ChIP-X experiments." Bioinformatics 26.19 (2010): 2438-2444.

[31] Kulakovskiy, Ivan V., et al. "HOCOMOCO: towards a complete collection of transcription factor binding models for human and mouse via large-scale ChIP-Seq analysis." Nucleic acids research 46.D1 (2018): D252-D259.

[32] Howe, Kevin L., et al. "Ensembl 2021." Nucleic acids research 49.D1 (2021): D884-D891.

[33] UniProt: the universal protein knowledgebase in 2021. Nucleic Acids Research, 2021, 49. Jg., Nr. D1, S. D480-D489.

[34] Kozomara, Ana, Maria Birgaoanu, and Sam Griffiths-Jones. "miRBase: from microRNA sequences to function." Nucleic acids research 47.D1 (2019): D155-D162.

[35] O'Leary, Nuala A., et al. "Reference sequence (RefSeq) database at NCBI: current status, taxonomic expansion, and functional annotation." Nucleic acids research 44.D1 (2016): D733-D745.

[36] Frankish, Adam, et al. "GENCODE reference annotation for the human and mouse genomes." Nucleic acids research 47.D1 (2019): D766-D773.

[37] Fishilevich, Simon, et al. "GeneHancer: genome-wide integration of enhancers and target genes in GeneCards." Database 2017 (2017).

[38] Nishimura, Darryl. "BioCarta." Biotech Software & Internet Report: The Computer Software Journal for Scient 2.3 (2001): 117-120.

[39] Sherry, Stephen T., et al. "dbSNP: the NCBI database of genetic variation." Nucleic acids research 29.1 (2001): 308-311.

[40] Schaefer, Carl F., et al. "PID: the pathway interaction database." Nucleic acids research 37.suppl_1 (2009): D674-D679.

[41] De, Supriyo, et al. "Disease and phenotype gene set analysis of disease-based gene expression in mouse and human." Physiological genomics 42.2 (2010): 162-167.

[42] Denny, Joshua C., et al. "Systematic comparison of phenome-wide association study of electronic medical record data and genome-wide association study data." Nature biotechnology 31.12 (2013): 1102-1111.

[43] Kitts, Paul A., et al. "Assembly: a resource for assembled genomes at NCBI." Nucleic acids research 44.D1 (2016): D73-D80.

[44] Thul, Peter J., and Cecilia Lindskog. "The human protein atlas: a spatial map of the human proteome." Protein Science 27.1 (2018): 233-244.

[45] Kheradpour, Pouya, and Manolis Kellis. "Systematic discovery and characterization of regulatory motifs in ENCODE TF binding experiments." Nucleic acids research 42.5 (2014): 2976-2987.

[46] Brown, Garth R., et al. "Gene: a gene-centered information resource at NCBI." Nucleic acids research 43.D1 (2015): D36-D42.

[47] Nurk, Sergey, et al. "The complete sequence of a human genome." bioRxiv (2021).

[48] Guennebaud, Gaël, et al. "Eigen v3" http://eigen.tuxfamily.org (2010)

[49] Tange, Ole. "Gnu parallel-the command-line power tool." The USENIX Magazine 36.1 (2011): 42-47.

[50] Bliek1ú, Christian, Pierre Bonami, and Andrea Lodi. "Solving mixed-integer quadratic programming problems with IBM-CPLEX: a progress report." Proceedings of the twenty-sixth RAMP symposium. 2014.

[51] Schäling, Boris. The boost C++ libraries. Boris Schäling, 2011.

[52] Vandenbrink, Joshua P., et al. "RNA-seq analyses of Arabidopsis thaliana seedlings after exposure to blue-light phototropic stimuli in microgravity." American journal of botany 106.11 (2019): 1466-1476.

[53] Herranz, Raúl, et al. "RNAseq analysis of the response of Arabidopsis thaliana to fractional gravity under blue-light stimulation during spaceflight." Frontiers in plant science 10 (2019): 1529.

[54] Krueger, Felix, et al. "FelixKrueger/TrimGalore: v0.6.7 - DOI via Zenodo (0.6.7)" Zenodo (2021): https://doi.org/10.5281/zenodo.5127899

[55] Dobin, Alexander, et al. "STAR: ultrafast universal RNA-seq aligner." Bioinformatics 29.1 (2013): 15-21.

[56] Li, Bo, and Colin N. Dewey. "RSEM: accurate transcript quantification from RNA-Seq data with or without a reference genome." BMC bioinformatics 12.1 (2011): 1-16.

[57] Love, Michael I., Wolfgang Huber, and Simon Anders. "Moderated estimation of fold change and dispersion for RNA-seq data with DESeq2." Genome biology 15.12 (2014): 1-21.

[58] Matía, Isabel, et al. "Plant cell proliferation and growth are altered by microgravity conditions in spaceflight." Journal of plant physiology 167.3 (2010): 184-193.

[59] Manzano, Ana Isabel, et al. "Meristematic cell proliferation and ribosome biogenesis are decoupled in diamagnetically levitated Arabidopsis seedlings." BMC plant biology 13.1 (2013): 1-15.

[60] Valbuena, Miguel A., et al. "The combined effects of real or simulated microgravity and red-light photoactivation on plant root meristematic cells." Planta 248.3 (2018): 691-704.

[61] Stutte, G. W., et al. "Microgravity effects on leaf morphology, cell structure, carbon metabolism and mRNA expression of dwarf wheat." Planta 224.5 (2006): 1038-1049.

[62] Adamchuk, N. I. "Ultrastructural and functional changes of photosynthetic apparatus of Arabidopsis thaliana (L.) Heynh induced by clinorotation." Advances in Space Research 21.8-9 (1998): 1131-1134.

[63] Miyamoto, K., et al. "Leaf senescence under various gravity conditions: relevance to the dynamics of plant hormones." Advances in Space Research 27.5 (2001): 1017-1022.

[64] Shi, Kui, et al. "Transcriptome and proteomic analyses reveal multiple differences associated with chloroplast development in the spaceflight-induced wheat albino mutant mta." PloS one 12.5 (2017): e0177992.
